# Return to Sports and Functional Outcomes after Autologous Platelet-Rich Fibrin Matrix (PRFM) and Debridement in Midportion Achilles Tendinopathy: A Case Series with 24-Month Follow-Up

**DOI:** 10.3390/jcm12072747

**Published:** 2023-04-06

**Authors:** Venanzio Iacono, Simone Natali, Luca De Berardinis, Daniele Screpis, Antonio Pompilio Gigante, Claudio Zorzi

**Affiliations:** 1 Department of Orthopaedics, IRCCS Ospedale Sacro Cuore Don Calabria, 37024 Negrar, Italy; 2 Clinical Orthopaedics, Department of Clinical and Molecular Sciences, Università Politecnica delle Marche, 60020 Ancona, Italy

**Keywords:** Achilles tendon, tendinopathy, return to sports, return to work, athletes, autologous PRFM

## Abstract

(1) Background: Achilles tendinopathy (AT) is characterized by load-induced tendon pain, stiffness, and functional impairment that may affect the tendon midportion or insertion. Platelet-rich fibrin matrix (PRFM) is a promising adjunctive therapy for AT. We analyzed 24-month pain and functional outcomes in a cohort of patients managed by tendon debridement and autologous PRFM application to determine whether the combined treatment ensured an early return to sports/work and satisfactory clinical outcomes and functional scores. (2) Methods: The 24-month outcomes of 32 sport-practicing patients with chronic midportion AT treated with debridement and autologous PRFM were evaluated in terms of time to return to sports/work. The AOFAS and VISA-A were computed preoperatively and at 6 and 24 months. Blazina scores were evaluated preoperatively and at 6 months; ankle range of motion was assessed at 1, 6, 12, 24 months; and patient satisfaction was assessed at 24 months. (3) Results: Altogether, all patients had resumed their sport(s) activity, at the same or higher level, after 25.41 days (±5.37). Regarding work, all patients were able to return to their jobs after 16.41 days (±2.43). Ankle dorsiflexion and plantarflexion increased significantly: the AOFAS rose from 54.56 (±6.47) to 97.06 (±4.06) and 98.88 (±2.21) at 6 and 12 months, respectively, and the mean VISA-A score rose from 69.16 (±7.35) preoperatively to 95.03 (±4.67) and 97.28 (±2.43) at 6 and 12 months, respectively, after treatment. There were no complications. Most (90.62%) patients were very satisfied. (4) Conclusions: In symptomatic midportion AT, surgical debridement and autologous PRFM ensured a fast return to sports/work (4 weeks), significantly improving AOFAS and VISA-A and Blazina scores already at 6 months and providing excellent clinical outcomes at 24 months.

## 1. Introduction

The Achilles tendon is the strongest and largest tendon in the human body and is subject to high tensile loads, particularly during running [1]. It is also the most commonly injured lower limb tendon, especially in sport-practicing individuals [2]. Achilles tendinopathy (AT) is characterized by load-induced tendon pain, stiffness, and functional impairment [3]. Microscopically, the disorder involves random tenocyte proliferation, disruption of collagen fibers, and consequent increase in non-collagenous matrix [4], whereas ultrasound imaging shows an altered structure and thickened, disorganized fibers [5]. Irrespective of their athletic prowess, individuals with AT report impaired performance [6], reduced participation in physical activity [7], and deficits in ankle joint plantarflexion strength and endurance [8]. AT is more frequent between the ages of 40 and 59 years [9] and affects both athletic and non-athletic populations [10,11], with a significant impact on health-related quality of life [12]. Several hypotheses have been advanced to explain AT pain; intratendinous degeneration (tendinosis), neurogenic inflammatory processes, and neovascularization all seem to play major roles [13]. AT involves the tendon midportion (55–65% of cases) or its insertion (20–25%) [14]. Midportion AT has been related to factors such as overuse, gender, endocrine disorders, chromosomal make-up, a high body mass index, metabolic factors, and poor vascularity [15]. The latter factor appears to play a large role [16]; indeed, the painful region coincides with the least vascularized tendon area [17], which is found 2–6 cm proximal to the tendon insertion onto the calcaneus [17]. Conservative treatments for midportion AT are numerous [18] and include eccentric exercises (which are still the gold standard) [19], load modification, orthoses, cryotherapy, massage, non-steroidal anti-inflammatory drugs, extracorporeal shockwave therapy, high-volume injections, and sclerotherapy [17]. Non-surgical management is effective in up to 75% of patients [18,20]. In the remaining cases, operative management is indicated [21]. Surgical treatments include excision of abnormal tissue in the tendon and paratenon; vascular disruption; tendon scarification to activate the regenerative process; and gastrocnemius recession to reduce tendon tension and overload. Transfer of an intact tendon (usually the flexor hallucis longus) can be performed in patients with poor Achilles tendon tissue [22].

In recent years, regenerative therapies utilizing biological augmentation have been increasingly investigated for their beneficial effects on degenerated tendons. This new branch of medicine involves the use of autologous or heterologous substances of the human body. Examples of these technologies includes: platelet-rich plasma, vascular fraction, and bone marrow concentration and adipose tissue stroma [23,24]. Autologous platelet biomaterials are an important source of chemical messengers widely used for regeneration in surgery. These cellular therapeutic technologies cover many pathologies, resulting in improvements in quality of life for patients [25].

Among the several available options to enhance tissue regeneration capacity, platelet-rich plasma (PRP)—a concentrate whose platelet content is greater than in the circulation—has been used as an adjunctive therapeutic strategy for skin and tendon healing for some years [26]. However, there is a lack of uniformity in the methods used to obtain it, including the possible use of bovine thrombin for activation [27]. Its mechanism of action has been related to its content in growth factors, including platelet-derived and insulin-like growth factor as well as transforming growth factor-beta [28]. A key feature of PRP is that it is autologous, which minimizes any side effects [29]. Platelet-rich fibrin matrix (PRFM), considered as a second-generation platelet concentrate, is obtained by centrifugation of autologous venous blood; it is rich in the soluble fibrinogen found in fibrin and has a tridimensional structure [30]. PRFM requires no additives such as bovine thrombin or anticoagulants. It consists of a matrix in which growth factors, cytokines, and platelets are retained and can continuously be released, supplying the necessary elements for wound healing and acting as a biodegradable scaffold for the delivery of factors that enhance cell growth, collagen synthesis, and angiogenesis. It is used in several branches of medicine [30]. We conducted this study to examine the long-term (24-month) pain, functional outcomes, and scores of a cohort of patients with AT who received tendon debridement and autologous PRFM augmentation. Our working hypothesis was that PRFM use would ensure an early return to sports and work as well as a satisfactory functional outcome.

## 2. Materials and Methods

### 2.1. Study Design

The records of the patients with midportion AT treated with debridement of abnormal tissue in the tendon and paratenon combined with autologous PRFM augmentation were retrieved from the institutional database. The data collected included demographics, side of operation, body mass index (BMI), tobacco use, sport(s) practiced, American Society of Anesthesiologists (ASA) class, operative time, and length of hospital stay. All procedures were performed by a single experienced surgeon (V.I.) at one institution. The study protocol was approved by the local Institutional Review Board. Informed consent was obtained from all patients. The study complies with the principles of the Helsinki Declaration.

On clinical assessment, all patients reported pain on the medial aspect of the Achilles midportion during activities involving tendon loading and tenderness on its ventromedial side.

### 2.2. Indication for Surgery

**Figure 1:**
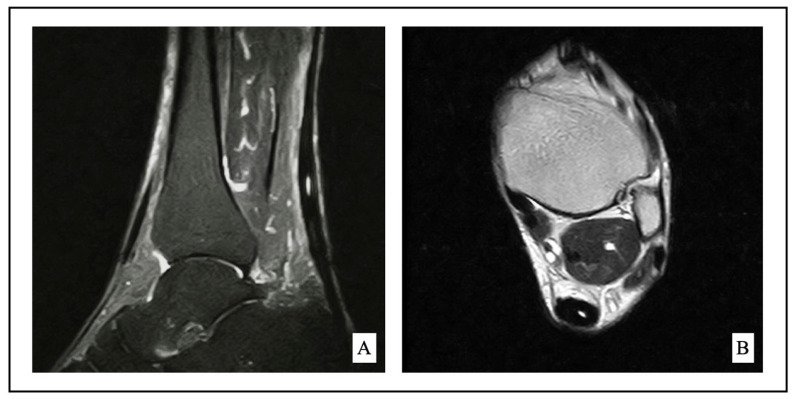
Sagittal fat-saturated (**A**) and axial T1 (**B**) show midportion Achilles tendinopathy.

The diagnosis of midportion AT was based on clinical history and magnetic resonance imaging (MRI) as shown in Figure 1. Failure of non-surgical treatment for at least 6 months was considered as an indication for surgical management [31]. Conservative treatment consisted of rest for at least 6 weeks, non-steroidal anti-inflammatory drugs, shockwave therapy, bracing, deep transverse friction massage, stretching, and eccentric strengthening prescribed by a physical therapist.

### 2.3. Inclusion and Exclusion Criteria

From August 2016 to October 2020, 83 consecutive patients with sport-related injuries (e.g., jogging, soccer, volleyball, and basketball) underwent Achilles tendon debridement and autologous PRFM augmentation at our institution. The inclusion criteria were age ≥ 18 years, pain involving the Achilles tendon midportion, midportion AT confirmed by MRI, and a follow-up of at least 24 months. We excluded elite athletes and patients aged > 40 years, those with chronic rupture, steroid injection-related injury, bony Achilles tendon avulsion, acute trauma more than 10 days previously, a history of neurological disorders, cortisone therapy, prior Achilles tendon surgery, a calcaneal slope > 30◦ (who are more suited to Zadek osteotomy), and those who had suffered a contralateral leg injury.

**Figure 2:**
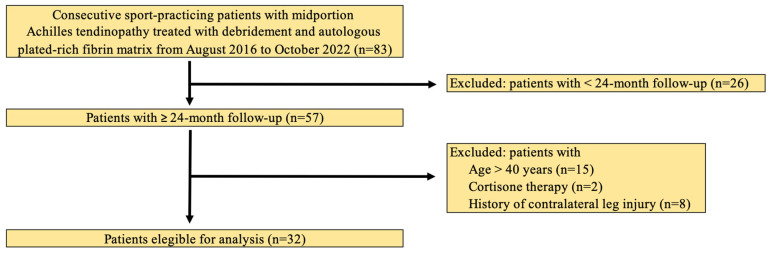
Patient eligibility.

Two-year follow-up data were available for 57 of these 83 patients. However, 15 were aged > 40 years, 2 were being treated with cortisone, and 8 had a history of contralateral leg injury. The flow diagram illustrating the patient selection process is reported in Figure 2.

### 2.4. Surgical Technique

**Figure 3:**
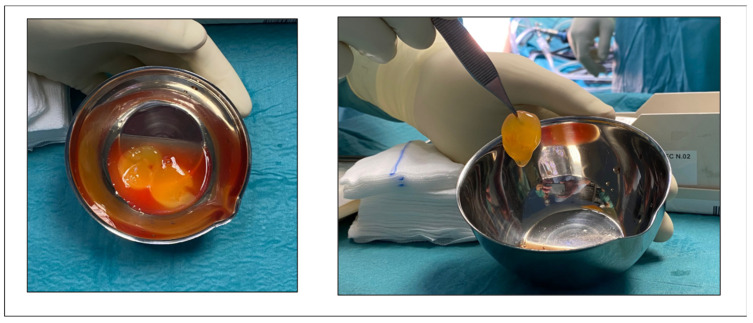
The autologous platelet-rich fibrin matrix (PRFM).

**Figure 4:**
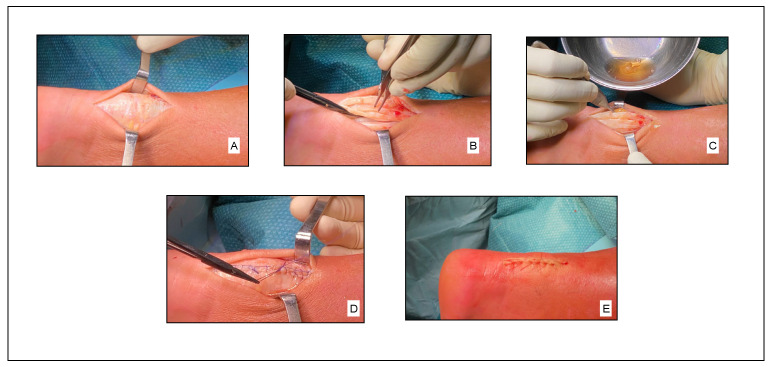
Surgical procedure: (**A**) access to the Achilles tendon; (**B**) debridement of the tendon midportion; (**C**) application of autologous PRFM; (**D**) suture of the fascia; (**E**) suture of the skin.

Patients, under general or spinal anesthesia, were placed in prone position. A pneumatic tourniquet was applied. Debridement of necrotic tissue, nodules, and any calcifications in the tendon and paratenon was performed using a midline approach. Autologous PRFM (Regen-kit Extracell Membrane, Regen Lab, Switzerland), obtained according to the manufacturer’s instructions following a standardized procedure (Figure 3), was applied to the treated tendon portion using absorbable sutures as shown in Figure 4.

### 2.5. Postoperative Rehabilitation

All patients followed the same postoperative rehabilitation protocol, consisting of a range of movement exercises, full weightbearing from day seven, and a structured strengthening program.

### 2.6. Preoperative Evaluation and Follow-Up

Preoperative and postoperative functional data were obtained retrospectively. Time to return to sports and work was determined. The VISA-A [32] and [33] the AOFAS were computed before the procedure and at 6 and 24 months to assess pain and function during daily activities. Blazina scores [33] were evaluated preoperatively and at 6 months to assess pain during sport practice. Ankle range of motion was evaluated in both limbs at 1, 6, 12, and 24 months by two of the authors (S.N. and L.D.B.) and the improvement computed as the difference between injured and uninjured extremity. Patient satisfaction (very satisfied; satisfied; not completely satisfied; dissatisfied) data were collected at 24 months. Any adverse events, e.g., infection, wound breakdown, requirements for further treatments(s), were recorded by the main investigator (S.N.).

### 2.7. Statistics

All analyses were performed using SPSS statistical software (SPSS vs 23.0, Chicago, IL, USA). Continuous variables were assessed using Student’s *t*-test for paired samples. The significance threshold was set at 0.05.

## 3. Results

### 3.1. Patient characteristics

After application of the inclusion and exclusion criteria, 32 patients were selected. Of the 32 patients, 46.88% were men and 53.13% were women. Mean age was 28.75 (±6.67) (range, 18.00–39.00). The most affected side was the left side in 56.25% of the patients. Mean BMI was 25.05 (±3.56) (range, 18.56–37.87); 53.13% were smokers. In addition, all patients practiced sports, in particular 31.25% tennis, 25.00% soccer, 6.25% basketball, 12.50% volleyball, 9.38% jogging, and 15.63% other sports. The mean operative time was 39.44 min (±7.84) (range, 21.00–55.00) and the mean of length of hospital stay was 1.16 days (±0.37) (range, 1.00–2.00). Demographic and preoperative details are reported in Table 1.

### 3.2. Return to Sports and Work

Altogether, all patients had resumed their sport(s) activity, at the same or higher level, after 25.41 days (±5.37) (range, 21.00–37.00). Regarding work, all patients were able to return to their jobs after 16.41 days (±2.43) (range, 14.00–22.00). All patients continued to play sports during the follow-up.

### 3.3. Ankle Range of Motion and Patient Satisfaction

The mean difference in ankle dorsiflexion in the injured limb compared with the uninjured limb was 3.19 (±0.74) (range, 2.00–4.00), 2.31 (±1.00) (range, 1.00–4.00), 1.66 (±0.83) (range, 1.00–3.00), and 1.22 (±0.42) (range, 1.00–2.00) at 1, 6, 12, and 24 months, respectively, whereas the mean difference in plantarflexion was 3.47 (±0.72) (range, 2.00–4.00), 2.47 (±0.51) (range, 2.00–3.00), 1.60 (±0.71) (range, 1.00–3.00), and 0.78 (±0.66) (range, 0.00–2.00), respectively. Patient satisfaction at 24 months was very high, with 90.62% of patients being very satisfied and 9.38% being satisfied with their outcome (Table 2).

### 3.4. AOFAS and VISA-A and Blazina Scores

The AOFAS and the VISA-A were computed before the procedure and at 6 and 24 months. Blazina scores were evaluated before the procedure and at 6 months.

The AOFAS rose from 54.56 (±6.47) (range, 42.00–65.00) to 97.06 (±4.06) (range, 87.00–100.00) (*p* < 0.01) at 6 months, with a mean improvement of 42.50 (±3.20) (range, 35.00–45.00). At 24 months, the AOFAS reached a value of 98.88 (±2.21) (range, 92.00–100.00), with a mean improvement of 44.31 (±5.16) (range, 35.00–54.00) compared to the preoperative value. The mean VISA-A scores rose from 69.16 (±7.35) (range, 56.00–81.00) to 95.03 (±4.67) (range, 85.00–100.00) at 6 months, with a mean improvement of 25.88 (±5.00) (range, 18.00–38.00) (*p* < 0.01). At 24 months, the mean VISA-A score was 97.28 (±2.43) (range, 93.00–100.00), with a mean improvement of 28.13 (±6.40) (range, 18.00–42.00) compared to the preoperative value. (Table 3).

**Figure 5:**
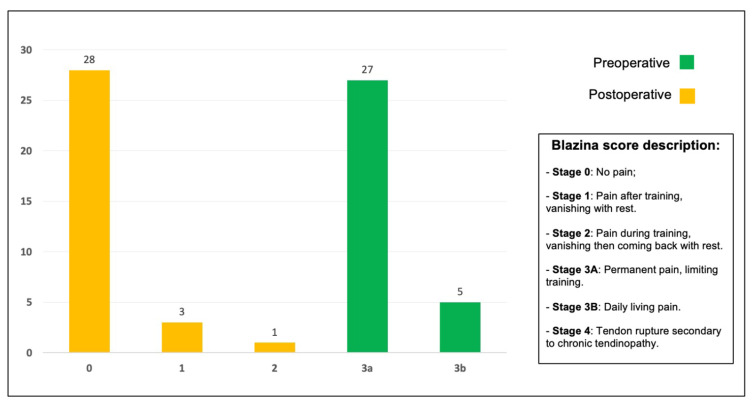
Blazina scores of the 32 patients before surgery and at 6 months.

Pain intensity was rated on a 0–3b Blazina score. The value before treatment was 3a in 27 patients and 3b in five patients, respectively indicating permanent pain which limits training and daily living pain. After 6 months following surgical treatment, 28 patients had no pain during training, three patients had pain after training which vanished with rest, and one patient had pain during training which vanished and then came back with rest. The Blazina scores computed before and after the procedure are shown in Figure 5.

### 3.5. Complications and Revisions

There were neither complications nor revisions.

## 4. Discussion

The goal of the study was to evaluate the return to sports and work, the clinical outcomes, and the functional scores of patients with midportion AT treated with debridement and autologous PRFM augmentation. The study findings confirm our hypothesis that the combined procedure provides an effective treatment for these patients. The treatment is also safe since none of the patients experienced infection, adverse effect, or recurrences during follow-up. Reports on the effect of PRP on tendon healing are numerous but contrasting.

In patients with chronic midportion AT, a single intratendinous PRP injection did not ameliorate tendon dysfunction at 6 months [34], while PRP combined with tendon debridement failed to improve outcomes compared with debridement alone [35]. A recent review and meta-analysis showed that in patients with chronic AT, PRP injection was more effective than a placebo in reducing pain at 6 weeks but not thereafter [36]. According to a systematic review, PRP injections appeared to improve patellar tendon healing but not AT, either as a conservative or as a surgical approach [26]. In other studies, PRP injections showed promising results as a conservative treatment. Monto et al. [37] described an AOFAS increase in AT patients managed with PRP monotherapy. Owens et al. [38] found a modest improvement in functional measures in patients with chronic midportion AT treated with PRP injection; however, the MRI appearance of the Achilles tendon remained unchanged during follow-up. Neither study had a control group. In a randomized controlled trial, Boesen et al. [39] demonstrated that, in chronic AT patients, PRP combined with eccentric training seemed more effective than eccentric training alone in improving activity levels, reducing pain and tendon thickness, and increasing intratendinous vascularity; however, a high-volume injection of saline, steroids, and local anesthetic appeared to induce better outcomes than PRP. Notably, none of these studies used autologous PRP. Some recent studies describe the use of PRFM in patients with AT. An imaging and histological study showed that a PRFM scaffold placed at the site of the tendon defect seemed to promote tissue healing of the Achilles tendon in rabbits [40].

In a rat study, PRFM improved and accelerated AT healing and repair compared with PRP [41]. In another comparative study, suturing and PRFM application after acute Achilles tendon rupture induced significant morphological modifications and functional improvements compared with suturing alone [42,43].

To the best of our knowledge, this is the first study describing autologous PRFM augmentation in sport-practicing individuals with chronic midportion AT. All patients achieved significantly improved clinical outcomes and functional scores compared with baseline and were satisfied or very satisfied. Moreover, none required conservative or surgical Achilles tendon treatment during follow-up. Notably, our patients return to sports after 25.41 (±5.37) days. This data should not be underestimated because in patients with symptomatic midportion AT, the current literature reports a return to sports of 14.10 (±5.20) weeks when patients were treated exclusively with surgical debridement [44].

Autologous PRFM is easy to apply, it has successfully been used to treat vascular ulcers, and has provided promising results in rotator cuff tears [45]. In our study, it was used as a “patch” to ameliorate the loss of tendon substance after debridement.

The limitations of the study include a small cohort and short follow-up; the fact that we did not measure the mechanical properties of the healed tendon, such as strain under loading and elastic modulus; and its non-randomized and non-controlled design, considering that, in particular, a non-PRFM group would have allowed investigation of the effectiveness of PRFM alone.

## 5. Conclusions

In conclusion, surgical management of symptomatic midportion AT with debridement and autologous PRFM application ensured a fast return to sports and work, it significantly increased AOFAS and VISA-A and Blazina scores at 6 months, and it provided excellent clinical outcomes at 24 months.

## Figures and Tables

**Table 1:** Preoperative and perioperative data.

Variable	Patients
Number	32.00
Age, mean (SD) [range]	28.75 (6.67) [18.00–39.00]
Gender	
Male (%)	15 (46.88)
Female (%)	17 (53.13)
Side:	
Right (%)	14 (43.75)
Left (%)	18 (56.25)
BMI (kg/m2), mean (SD) [range]	25.05 (3.56) [18.56–37.87]
Tobacco use (%)	17 (53.13)
Sport	
Tennis (%)	10 (31.25)
Soccer (%)	8 (25.00)
Basketball (%)	2 (6.25)
Volleyball (%)	4 (12.50)
Jogging (%)	3 (9.38)
Other sports (%)	5 (15.63)
ASA class	
ASA 1 (%)	24 (75.00)
ASA 2 (%)	8 (25.00)
Operative time (min), mean (SD) [range]	39.44 (7.84) [21.00–55.00]
Length of hospital stay (days), mean (SD) [range]	1.16 (0.37) [1.00–2.00]

SD: standard deviation; BMI: body mass index; ASA: American Society of Anesthesiology.

**Table 2:** Postoperative functional tests and patient satisfaction.

Variable	Patients (*n* = 32)
Difference in dorsiflexion between injured and uninjured ankle	
1 month, mean (SD) [range]	3.19 (0.74) [2.00–4.00]
6 months, mean (SD) [range]	2.31 (1.00) [1.00–4.00]
12 months, mean (SD) [range]	1.66 (0.83) [1.00–3.00]
24 months, mean (SD) [range]	1.22 (0.42) [1.00–2.00]
Difference in plantarflexion between injured and uninjured ankle	
1 month, mean (SD) [range]	3.47 (0.72) [2.00–4.00]
6 months, mean (SD) [range]	2.47 (0.51) [2.00–3.00]
12 months, mean (SD) [range]	1.60 (0.71) [1.00–3.00]
24 months, mean (SD) [range]	0.78 (0.66) [0.00–2.00]
Satisfaction at 24 months	
Very satisfied (%)	29 (90.62)
Satisfied (%)	3 (9.38)

SD: standard deviation.

**Table 3:** AOFAS and VISA-A scores before the procedure and at 6 and 24 months.

	Preoperative	Postoperative (6 Months)	Postoperative (24 Months)	*p*-Value
	Mean (SD) [Range]	Mean (SD) [Range]	Mean (SD) [Range]	
AOFAS	54.56 (6.47)	97.06 (4.06)	98.88 (2.21)	<0.01
	[42.00–65.00]	[87.00–100.00]	[92.00–100.00]	
Difference between score at 6 months and preoperative score; mean (SD) [range]	42.50 (3.20) [35.00–45.00]			
Difference between score at 24 months and preoperative score; mean (SD) [range]	44.31 (5.16) [35.00–54.00]			
VISA-A	69.16 (7.35)	95.03 (4.67)	97.28 (2.43)	<0.01
	[56.00–81.00]	[85.00–100.00]	[93.00–100.00]	
Difference between score at 6 months and preoperative score; mean (SD) [range]	25.88 (5.00) [18.00–38.00]			
Difference between score at 24 months and preoperative score; mean (SD) [range]	28.13 (6.40) [18.00–42.00]			

AOFAS: American Orthopedic Foot and Ankle Society Score; VISA-A: Victorian Institute of Sports Assessment-Achilles.

## Data Availability

The datasets generated during the current study are available from the corresponding author on reasonable request.
